# A multi-view validation framework for LLM-generated knowledge graphs of chronic kidney disease

**DOI:** 10.1007/s11548-025-03495-x

**Published:** 2025-08-14

**Authors:** Aditya Kumar, Dilpreet Singh, Mario Cypko, Oliver Amft

**Affiliations:** 1 Hahn-Schickard, 79110 Freiburg, Germany; 2 Intelligent Embedded Systems Lab, University of Freiburg, 79110 Freiburg, Germany

**Keywords:** Knowledge graphs, Triples, Chronic Kidney Disease, Knowledge-driven modelling, LLMs, Validation, Expert system, Semantic evaluation, Model-guided medicine, medical informatics, Healthcare, Nephrology

## Abstract

**Purpose:**

The goal of our work is to develop a multi-view validation framework for evaluating LLM-generated knowledge graph (KG) triples. The proposed approach aims to address the lack of established validation procedure in the context of LLM-supported KG construction.

**Methods:**

The proposed framework evaluates the LLM-generated triples across three dimensions: semantic plausibility, ontology-grounded type compatibility, and structural importance. We demonstrate the performance for GPT-4 generated concept-specific (e.g., for medications, diagnosis, procedures) triples in the context of chronic kidney disease (CKD).

**Results:**

The proposed approach consistently achieves high-quality results across evaluated GPT-4 generated triples, strong semantic plausibility (semantic score mean: 0.79), excellent type compatibility (type score mean: 0.84), and high structural importance of entities within the CKD knowledge domain (ResourceRank mean: 0.94).

**Conclusion:**

The validation framework offers a reliable and scalable method for evaluating quality and validity of LLM-generated triples across three views: semantic plausibility, type compatibility, and structural importance. The framework demonstrates robust performance in filtering high-quality triples and lays a strong foundation for fast and reliable medical KG construction and validation.

## Introduction

Knowledge graphs (KGs) have emerged as a transformative tool in health care, offering a structured representation of complex and interconnected medical knowledge [1]. KGs represent medical concepts (e.g., diagnosis, procedure, medications). At the core of KGs are triples, which consist of a head, relation, and tail (e.g., *“Hypertension”—“is treated with”—“ACE inhibitors”*) [2]. Triples encapsulate semantic relationships between entities to facilitate advanced applications, including clinical decision support, disease modeling, and personalized care [3, 4]. However, creating KGs directly from electronic health records (EHRs) often involves only simple relationships, e.g., basic hierarchies or co-occurrence links [5–7]. Furthermore, KGs built only on EHRs tend to overlook rich clinical knowledge available in curated biomedical resources, including UMLS [8] and SNOMED-CT [9]. As a result, KGs built from EHR data typically fail to capture deeper and aggregated representations of medical concepts. For example, the relationship of chronic kidney disease stage with hyperparathyroidism as co-condition cannot be directly derived from EHR without additional clinical knowledge. Moreover, building KGs remains a time-intensive, manual process heavily reliant on expert curation, which limits the scalability and rapid deployment of KGs in healthcare contexts [10, 11].

The advent of large language models (LLMs), including GPT-4 [12], presents an opportunity to accelerate KG development. With the ability of LLMs to extract semantic relationships and generate knowledge triples, LLMs hold promise for automating large portions of the KG construction process [13]. Previous works have demonstrated the potential of LLMs in aiding KG construction [14–16]. By leveraging LLM-generated triples, the time and complexity associated with expert curation could be profoundly reduced and focused on more complex triples and essential tasks where domain expertise is indispensable. Recent works have demonstrated that it is possible to extract KG triples using LLMs, where the focus is to derive concept-specific knowledge, e.g., triples for medication. Arsenyan et al. [17] have demonstrated that LLMs, including GPT variants, can extract concept-specific triples from unstructured notes in EHRs using tailored prompts. Yang et al. [18] used GPT-4 to build a comprehensive sepsis KG by extracting concept-specific triples from both unstructured notes and structured tables with evaluation of model accuracy and clinical relevance. Gonzalez et al. [19] built a gene-disease KG using a BioBERT pipeline with entity normalization and ontology alignment. However, a major limitation in the context of LLM-generated triples is the lack of a robust validation framework. Some studies include basic validation, e.g., model performance, expert review, and ontology lookup; however, there is a need for a comprehensive validation framework that includes semantic plausibility, ontology-grounded checks on logic and structural importance. The absence of an established validation procedure raises concerns about the reliability of the extracted triples, as errors or inconsistencies in medical KGs have serious implications for downstream applications [20].

**Fig. 1:**
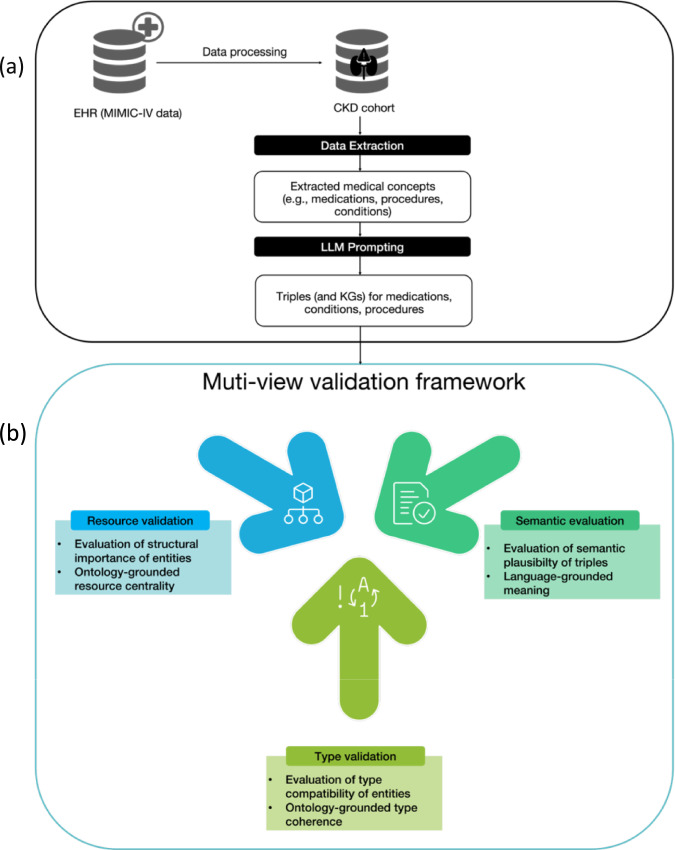
Overview of our triple generation and validation framework. **a** Triple generation process in the context of chronic kidney disease (CKD). Medical concepts were extracted from structured data, LLM prompts were used to generate concept-specific triples that were subsequently aggregated to concept-specific KGs. **b** Our multi-view validation framework for evaluating LLM-generated triples across the three dimensions: semantic plausibility, ontology-grounded type compatibility, and structural importance

In this work, we propose a multi-view validation framework for LLM-generated triples across three complimentary dimensions: semantic plausibility, ontology-grounded type compatibility, and structural importance. The framework assesses triples through semantic evaluation to ensure semantic plausibility, type validation to verify that entities (i.e., head/tail of triples) adhere to ontology-guided type constraints, and resource validation to assess how structurally important an entity is within the established clinical knowledge. In combination, the aforementioned dimensions balance data-driven, domain-driven, and topology-driven evidence to ensure triple quality and validity. We demonstrate the framework's performance on GPT-4 generated concept-specific triples of chronic kidney disease (CKD). Figure 1 illustrates the triple generation process and the multi-view validation framework.

## Methods

### Data and pre-processing

MIMIC-IV v2.2 dataset [21] was used for this study and a pre-processing pipeline developed by Gupta et al. [22] was utilized to extract a corpus of patients diagnosed with CKD, which we refer as the CKD cohort. We then extracted medical concepts, i.e., unique terms from the CKD cohort including medications, conditions, and procedures, including CKD-related comorbidities.

### Extraction of triples using LLM

We used GPT-4 to generate the triples. Triples were derived for medical concepts derived in the previous step. The extracted medical concepts were used as input to GPT-4 with a structured prompt, as suggested by Jiang et al. [13]. The prompt was designed to generate directed triples in the format [[entity1, relationship, entity2]]. GPT-4 generated concept-specific triples that were then aggregated to form a KG for each medical concept.

### Semantic evaluation

To assess the semantic plausibility of triples generated by GPT-4, we implemented a semantic evaluation framework. We adapted the translation energy function (TEF) [23] to the medical domain by incorporating PubMedBERT [24] embeddings, in place of traditional TransE [23] embeddings. Each triple element, head (*h*), relation (*r*), and tail (*t*) was converted to a vector using PubMedBERT. The idea is, a triple (*h*, *r*, *t*) is semantically plausible if *h* + *r*≈*t*. Euclidean distance, ${d}_{\text{trans}}={\left|\left|h+r-t\right|\right|}^{2}$ with the vectors *h* + *r* and *t* was calculated, which was quadratically penalized, i.e., large deviations were penalized more. For the final semantic plausibility score, we normalized the distances to a [0,1] scale using min–max normalization across all triples. The scores were reported as mean, the average semantic plausibility across all LLM-generated triples and standard deviation, showing how much variation exists in semantic plausibility across all triples.

### Type validation

To ensure compatibility between entities or type coherence in GPT-4-generated triples, we implemented an enhanced type validation framework [25] leveraging the CKD ontology^1^ [26]. The CKD ontology is a domain specific ontology derived from primary care data and clinical coding standards. CKD ontology is designed to identify the stage of CKD based on estimated globular filtration rate (eGFR), proteinuria, and diagnostic codes. CKD ontology serves as a trusted, evidence-based source of ground truth due to its guideline-aligned construction.

The type validation framework systematically verifies the compatibility of medical concepts by utilizing CKD ontology’s hierarchical structure. For each head and tail concept in a triple, the semantic type was extracted from the CKD ontology node. Graph traversal up the CKD ontology was performed to include all ancestor types. The ancestors represent generalized semantic categories and create a type set for each entity. Compatibility between entities was assessed using Jaccard similarity [27]:

$$\text{Jaccard}\left({T}_{\text{head}},{T}_{\text{tail}}\right)=\frac{\left|{T}_{\text{head}}\cap {T}_{\text{tail}}\right|}{\left|{T}_{\text{head}}\cup {T}_{\text{tail}}\right|},$$

where *T*_head_ and *T*_tail_ are sets of semantic types and ancestors for head and tail.

Jaccard similarity represents the intersection over the union of the respective type sets (i.e., semantic relationships) to ensure adherence to the semantic structure defined by the CKD ontology. If an entity type was not found in the CKD ontology, a fallback mechanism based on PubMedBERT embedding similarity was used to evaluate compatibility. In detail, PubMedBERT embeddings were used to represent the concept and then derive the cosine similarity between head and tail vectors as proxy for semantic type compatibility. The hybrid approach combining ontology-based validation and embedding-driven fallback provides a robust mechanism for verifying the type coherence of triples in the medical knowledge graph. The type score was summarized by the normalized mean and standard deviation across all triples.

### Resource validation

We designed a resource validation framework leveraging the connectivity patterns and hierarchical organization of the CKD ontology. The framework evaluates the structural importance of an entity by analyzing the position within the CKD ontology graph and the structural connections to other entities. Each entity (head and tail) in a triple was matched to a node in CKD ontology. If no match was found, the tripe was flagged. Direct (e.g., A → B) and indirect (e.g., A → X → B) relationships were assessed through graph connectivity patterns, while structural relationships including parent, child, or sibling roles within the hierarchy are used to confirm the plausibility of triples. To quantify the importance and reliability of entities, we implemented ResourceRank [23]:

$$\text{R}\left({v}_{i}\right)=\left(1-d\right)+\text{d}{\sum }_{{v}_{j}\in In\left({v}_{i}\right)}\frac{R\left({v}_{j}\right)}{\left|\text{Out}\left({v}_{j}\right)\right|},$$

where *d* $\in$ (0,1) is the damping factor (in our case set to 0.85), In(*v*_*i*_) = set of nodes that link to *v*_*i*_, Out(*v*_*j*_) = number of outgoing edges from *v*_*j*_.

ResourceRank quantifies how central and well-connected an entity is within the CKD ontology, considering both the number of entities that link to the entity and the importance of the linking entities. The ResourceRank score was computed for each entity to flag rare, isolated, or poorly connected entities. A triple was considered resource valid if the head and tail are connected via plausible paths in the ontology, and position/role in the hierarchy makes the triple conceptually consistent. The ResourceRank was also reported as normalized mean and standard deviation for all triples.

## Results

**Fig. 2:**
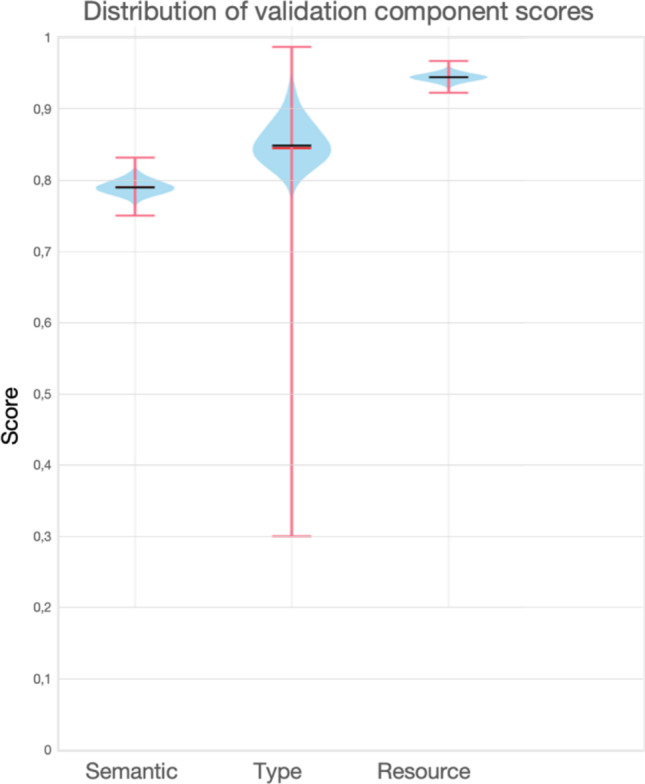
Distribution of components of our validation framework. The distribution of scores across the validation components: semantic score, type score, and ResourceRank. Semantic score (mean: 0.79, standard deviation: 0.01) shows good semantic plausibility. Type score (mean: 0.84, standard deviation: 0.07) shows some variability due to less-represented entities. ResourceRank (mean: 0.94, standard deviation: 0.005) exhibits strong connectivity

Our validation framework applied to GPT-4-generated triples produced consistent results across multiple components. Figure 2 illustrates the distribution of the components of our validation framework.

The semantic score had a mean of 0.79 across all triples (standard deviation: 0.01, range: [0.75, 0.83], indicating strong semantic plausibility of the triples. The semantic score reflects the degree to which the triple conforms to translational plausibility in the embedding space. Higher scores (> 0.75) indicate that the triple is semantically plausible (*h* + *r*≈*t*), while scores close to zero indicate semantic mismatch.

The type score showed a mean of 0.84 across all triples (standard deviation: 0.07, range: [0.30, 0.98]), indicating good overall type compatibility between entities with some variability for entities less represented in the ontology. The type score measures whether the entities in a triple are structurally and semantically compatible based on the ontology-derived type sets corresponding to the entity. A higher score (> 0.75) implies strong type coherence, while a lower score may indicate conceptual mismatch. In our case, the reported standard deviation of 0.07 was slightly higher than what can be considered good (< 0.05), which may have been a result of variability for entities that are not fully represented in the CKD ontology.

The ResourceRank had a mean of 0.94 across all triples (standard deviation: 0.005, range: [0.925, 0.966]), indicating that the entities in triples are structurally important in the ontology. ResourceRank evaluates how central and well-connected an entity is to trusted clinical knowledge (coming from ontology). A higher score (> 0.75) reflects stronger ontological grounding. In our case, the score achieved can be considered excellent (mean substantially higher than 0.75 and standard deviation profoundly lower than 0.05), which demonstrates strong alignment with trusted ontology-based medical knowledge with extremely low variability.

## Discussion

The proposed multi-view validation framework demonstrated strong performance, with high mean scores across all triples (mean scores > 0.75) and consistently low standard deviations (< 0.05) for all components. The three dimensions, semantic plausibility, type compatibility, and structural importance collectively offer complimentary insights and provide a strong and robust foundation for assessing triple quality and validity.

However, certain limitations remain. While our framework addresses core aspects of triple quality and validity, certain dimensions remain unaddressed. Temporal validity (i.e., distinguishing current vs. outdated relationships), relationship strength or probability, and explicit contradiction detection are not captured by the current approach. Future work will address more dimensions of triple quality and validity.

Currently our framework focuses on individual triple quality rather than collective knowledge coverage, i.e., the framework does not assess triple diversity. In this work, we prioritized clinical relevance and semantic alignment rather than analyzing the breadth or coverage of the KG across medical domains and concepts. Measuring diversity, including concept coverage, relation variability, and entropy, remains an open challenge. Moreover, optimizing diversity in parallel to clinical relevance (e.g., rejecting questionable triples) and semantic alignment is a problem left for future research. Nevertheless, in its current form, our framework already provides important support for multi-view KG optimization.

Furthermore, limited access to established medical knowledge graphs beyond the CKD ontology restricts cross-graph validation. Expert evaluations would provide valuable additional validation, particularly for complex triples, despite the potential for inconsistencies due to subjective biases and varying expert opinions.

Despite the limitations, the framework’s consistently low standard deviations (< 0.05) across components demonstrate reliable assessment capabilities. Ongoing efforts will address current limitations by introducing rule-based, domain knowledge-aware checks to classify and filter triples as general or medically relevant. The rules will be derived from established medical ontologies, such as the CKD ontology, and expert-curated guidelines, focusing on verifying the logical relationships between entities (e.g., ensuring drugs are appropriately linked to treatments or conditions). Additionally, external resources, e.g., the UMLS will be leveraged for cross-validation to enhance the reliability of the classifications. Furthermore, contrastive learning approaches will be integrated to refine semantic evaluation by improving the model’s ability to discern subtle contextual differences between triples.

## Conclusion

Our proposed multi-view validation framework provides a reliable and scalable method to evaluate quality and validity of LLM-generated triples. The framework assesses the triples across three complementary dimensions: semantic plausibility, type compatibility, and structural importance. High mean scores (> 0.75) and low standard deviations (< 0.05) across the three dimensions highlight its robustness. Further work will address more dimensions of triple quality and validity as well as an evaluation of triple diversity. In its current form, our framework lays a strong foundation for fast and reliable construction and validation of medical KGs.

## Data Availability

MIMIC-IV version 2.2 is available under restricted access via the PhysioNet platform [28], where applicants must complete a data use agreement and fulfill necessary requirements for access approval, including training on human subjects research.
